# The impact of sex, gender and pregnancy on 2009 H1N1 disease

**DOI:** 10.1186/2042-6410-1-5

**Published:** 2010-11-04

**Authors:** Sabra L Klein, Catherine Passaretti, Martha Anker, Peju Olukoya, Andrew Pekosz

**Affiliations:** 1 W. Harry Feinstone Department of Molecular Microbiology and Immunology, The Johns Hopkins Bloomberg School of Public Health, Baltimore, Maryland, USA; 2 Department of Biochemistry and Molecular Biology, The Johns Hopkins Bloomberg School of Public Health, Baltimore, Maryland, USA; 3 Department of Medicine, Infectious Diseases Division, The Johns Hopkins School of Medicine, Baltimore, Maryland, USA; 4 Division of Biostatistics and Epidemiology, University of Massachusetts School of Public Health and Health Sciences, Amherst, Massachusetts, USA; 5 Department of Gender, Women & Health, World Health Organization, Geneva, Switzerland

## Abstract

Children and young adults of reproductive age have emerged as groups that are highly vulnerable to the current 2009 H1N1 pandemic. The sex of an individual is a fundamental factor that can influence exposure, susceptibility and immune responses to influenza. Worldwide, the incidence, disease burden, morbidity and mortality rates following exposure to the 2009 H1N1 influenza virus differ between males and females and are often age-dependent. Pregnancy and differences in the presentation of various risk factors contribute to the worse outcome of infection in women. Vaccination and antiviral treatment efficacy also vary in a sex-dependent manner. Finally, sex-specific genetic and hormonal differences may contribute to the severity of influenza and the clearance of viral infection. The contribution of sex and gender to influenza can only be determined by a greater consideration of these factors in clinical and epidemiological studies and increased research into the biological basis underlying these differences.

## Sex, gender and pregnancy in the 2009 H1N1 pandemic

Sex and gender differences can affect exposure to pathogens, vulnerability to infectious diseases, health seeking behaviours and immune responses to pathogens, resulting in differences between males and females in the incidence, duration, severity and case fatality rates following an infection [1,2]. **Sex **refers to the biological and physiological characteristics that define males and females, whereas **gender **refers to the roles, behaviours, activities and attributes that individual societies consider appropriate for men and women. The impact of sex and gender on infection is tied to the age of the individual, as both biological and cultural factors can change dramatically with age. Consideration of these factors can result in a more effective public health response to infectious diseases, including influenza, and yet they are often inadequately addressed in clinical and basic research studies. A systematic review of the literature regarding sex, gender, pregnancy and the 2009 H1N1 pandemic indicates these are important factors which alter the severity of the disease as well as the prevention and treatment measures. A greater awareness of how sex and gender impact upon the biology of 2009 H1N1 infection could provide important insights into the unique morbidity and mortality patterns associated with this pandemic.

## 2009 H1N1 Overview

### Virus

An influenza pandemic was declared by the World Health Organization (WHO) in June 2009 and the virus, 2009 H1N1, became the primary influenza virus strain isolated from humans by the end of the winter influenza season in the southern hemisphere [3]. It was the dominant influenza A virus strain circulating in the northern hemisphere for the entire influenza season, effectively outcompeting both seasonal influenza A virus strains [3].

### Biological factors associated with severe 2009 H1N1 infection

The pandemic has been termed mild due to the relatively low mortality. Confirmed influenza virus infections, however, have increased substantially compared to recent years and the US Center for Disease Control (CDC) estimates of the number of people infected with 2009 H1N1 are greater than what would be expected in a standard influenza season [3].

#### Younger age

Most cases of severe disease and mortality after infection with seasonal influenza A virus occur in the ≥65 years population. In contrast, 2009 H1N1 has not been associated with a large number of infections in this age group but has the highest attack and hospitalization rates in individuals between the ages of 0-40. The reduced number of cases in those aged ≥65 stems in part from the fact that antibodies generated to pre-1950 H1N1 viruses cross react with 2009 H1N1, resulting in limited protection from 2009 H1N1 infection [3].

#### Presence of co-morbidities or risk factors for severe disease

Several populations are at risk for severe disease from seasonal as well as 2009 H1N1 infection [4], including individuals who have pre-existing illnesses or medical conditions, pregnant women, immunosuppressed individuals (either through treatment, HIV infection, or as a result of a pre-existing immunosuppressive disorder) and children aged 0-4 years. Medical conditions associated with an increased risk of severe disease include chronic respiratory disorders (for example, asthma, bronchitis, chronic obstructive pulmonary disease [COPD] and cystic fibrosis), neuromuscular disorders (for example, cerebral palsy, myasthenia gravis and muscular dystrophy), metabolic diseases (for example, diabetes) and chronic renal, heart or liver disorders [3]. Factors such as obesity and hypertension are not normally associated with severe disease from seasonal influenza but have been suggested as risk factors for severe disease from 2009 H1N1 in some studies [5-7].

#### Host immune responses

The protective immunity induced by influenza vaccinations is mediated primarily by antibodies that recognize the viral haemagglutinin protein and neutralize virus infectivity. After virus infection, host innate immune responses, including production of cytokines and chemokines, are activated which initiate a cascade of immunological events that lead to the development of specific immune responses to the virus. Controlling and clearing influenza virus infection requires neutralizing antibodies and cell-mediated immunity (for example, activation of T cells) [3]. The influx of immune cells into an influenza-infected lung can lead to the overproduction of various cytokines and chemokines - often called a 'cytokine storm' - which can enhance the virus-induced lung damage resulting in severe illness. A limited number of studies suggest that an altered cytokine and chemokine response is contributing to severe 2009 H1N1 disease [8,9]. Therefore, immunity to influenza viruses represents a balance between immune responses inducing protection and clearance of virus versus causing pathology.

### Male-female differences in 2009 H1N1-related morbidity and mortality

Utilizing published observational reports of patients with confirmed 2009 H1N1 infection and those admitted into intensive care units worldwide, the incidence, severity and case fatality rates following infection appear to differ between males and females, but often are age-dependent and vary between countries. The outcome of infection with 2009 H1N1 is generally worse for females, but the magnitude of this difference varies across geographical regions.

#### Incidence

Assessments of male-female differences in reported incidences of infection is confounded by two factors: (1) many countries do not disaggregate data by both sex and age which may mask sex differences among the age groups that are most likely to be exposed - children and young adults; and (2) the profound differences in health seeking behaviours between males and females [10].

Household transmission studies of children and adults reveal that being female (female relative risk [RR]: 1.87, 95% confidence interval [CI]: 1.17-2.73) is a significant factor associated with higher secondary attack rates of influenza-like illness, with attack rates being higher among children and young adults than older adults (>55 years of age) [11]. Reported male-female differences in the incidence of infection vary with age in several countries, with a higher incidence of infection with 2009 H1N1 in young women than young men of comparable age [12-15]. While pregnancy has been clearly linked with increased disease severity, the vast majority of infected females of reproductive ages are not pregnant, suggesting that additional factors are contributing to the increased incidence of infection. In contrast, in Asia, the majority of reported H1N1 cases have been male (57.1%) [16,17]. In China, males (male odds ratio [OR]: 1.94, 95% CI: 1.07-2.66) also shed the 2009 H1N1 virus in pharyngeal and nasopharyngeal samples for a longer duration than females [18] suggesting that the transmission potential may be higher in males. Other countries reported no male-female differences in the number of cases of 2009 H1N1, but did not analyse the data stratified by both age and sex [19-25].

#### Morbidity

**Figure 1:**
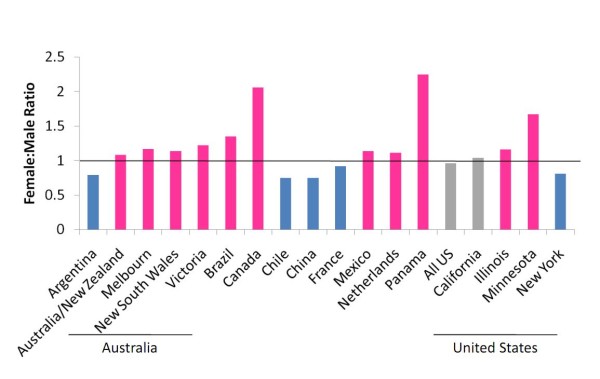
**Rates of hospitalization with severe 2009 H1N1 are higher among females than males in a majority of published datasets**. Female to male ratios of hospitalization with confirmed 2009 H1N1 were calculated using published datasets [5,6,14,16,27-30,32-37,43,134-136]. Pink bars = higher rates of hospitalization in females; blue bars = higher rates of hospitalization in males; grey bars = similar hospitalization rates in males and females. Details about sample sizes, time of data collection, and criteria for hospitalization are contained within each individual reference.

One trend that appears consistent across more than 60% of the datasets evaluated is that more females are hospitalized with critical illness than males (Figure 1). The first cases in the USA were in California (April-May 2009), where the a majority of hospitalized cases (21/26) were women, five of whom were pregnant [26]. Initial analyses of data from critically ill patients in the USA during the first wave reported no male-female difference [27], but subsequent state-specific reports from the first and second waves illustrated differences between the sexes [28-30]. In Canada, a significant majority of critically ill patients have been young women (female RR: 1.3, 95% CI: 1.0-1.6) [5,31]. Other countries also report that rates of hospitalization have been higher among females than males, with a majority of the females being of reproductive age (15-49 years of age) [14,32-36]. Analyses of cohorts of patients in Mexico and Australia/New Zealand revealed a trend for more females than males being hospitalized [6,37]. Evaluation of these differences in some countries is confounded by age, as many studies do not report male-female differences according to age group [6,27,37].

An examination of sex differences disaggregated by age is needed in larger, more complete datasets. The reason for the greater proportion of hospitalized women is not known, but many cases involve co-morbid conditions, including chronic respiratory diseases (for example. asthma and COPD), which are often more severe in females [38-40].

#### Mortality

Mortality from 2009 H1N1 is not common but data from South Africa, where the incidence of co-infection with HIV and tuberculosis is high, reveal that 65% of fatal cases were females of reproductive age, of whom almost half were pregnant [41]. RR of death is higher for young adult women (female RR: 1.5, 95% CI: 0.9-2.3) than men in Canada [31]. In Australia, 58% of fatal cases were male [35] and in Brazil and Peru case fatality rates have been equal between males and females [14,20]. No consistent pattern of male-female differences in mortality from the 2009 H1N1 has emerged.

### Effects of pregnancy on the severity of 2009 H1N1 disease

Increased morbidity and mortality in pregnant women has been documented during influenza pandemics and influenza seasons where virus infection rates are particularly high [42].

#### Morbidity and mortality

**Figure 2:**
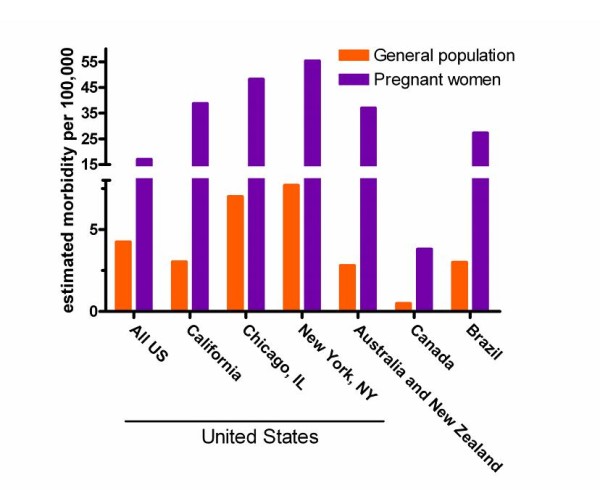
**Rates of severe influenza disease among pregnant women and the general population**. Estimated morbidity rates from April to December, 2009 for the 2009 H1N1 pandemic in select countries or geographic regions. Morbidity estimates are calculated based on datasets for the USA [27], Chicago, IL, USA [28], California, USA [43], New York, USA [49], Australia and New Zealand [37], Canada [5] and Brazil [14]. Estimates of the general population and pregnant woman are based on data from the US Census Bureau or the World Health Organization.

Pregnant women represent a disproportionately higher percentage of severe cases with the increased risk ranging from four- to 10-fold greater compared with the general population (Figure 2). Increased morbidity and mortality in pregnant women has been reported in many datasets [5,14,27,28,36,37,41,43-45]. The disease course and clinical presentation [46-48] has been studied and comparisons of disease in pregnant women to age-matched non-pregnant women [37,49,50] or to the general population [51] have been made. Disease severity is increased during the second and third trimester. However, no clear clinical parameter has been associated with pregnancy-associated increased morbidity and mortality. There are no significant differences in the general symptoms of disease, the progression to viral pneumonia, acute respiratory distress syndrome (ARDS) or secondary bacterial pneumonia in pregnant women compared to the control populations. Severe disease was also associated with a greater than sixfold increase in adverse neonatal outcomes when compared with pregnant women suffering mild disease [49]. An increased risk of severe disease may be present during the early postpartum period but the reported number of cases is limited and requires additional investigation [37,50].

#### Risk factors

**Figure 3:**
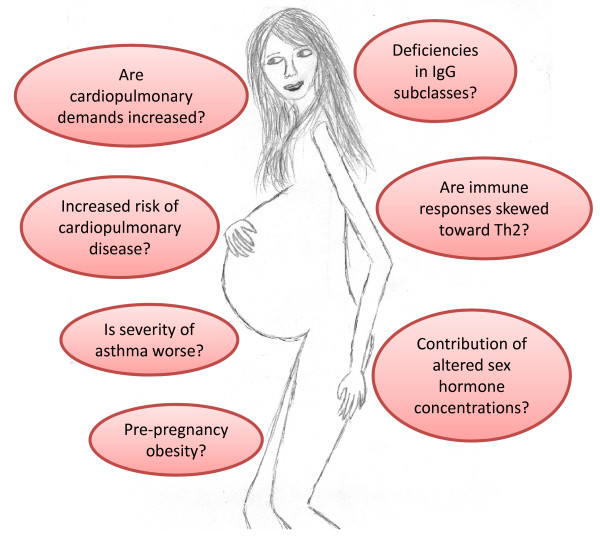
**Why are pregnant women at increased risk for severe 2009 H1N1 disease? **While 2009 H1N1 infection results in increased disease severity in pregnant women, the precise mechanisms responsible for this risk are not yet defined. The contribution of multiple biological factors to disease severity needs to be more thoroughly investigated.

Pregnancy itself is considered a risk factor for severe disease [3] but the biological basis for this has not been established. Pregnancy-associated changes in immune function, hormone levels, cardiopulmonary stress and difficulties in treatment for respiratory disease are often cited as important factors [52]. The presence of other risk factors may increase the risk of severe disease in a pregnant woman (Figure 3). The presence of a known co-morbidity in pregnant women with severe 2009 H1N1 disease can vary greatly and has been documented as 16% to 56% [37,46-50,53]. There is no one or cluster of co-morbidities associated with increased disease severity in pregnant women. Data indicate that when no additional co-morbidities were present, pregnant women still had a seven- to tenfold higher rate of severe disease when compared to age-matched, non-pregnant women [49,50].

### Male-female differences in risk factors for severe 2009 H1N1 disease

**Figure 4:**
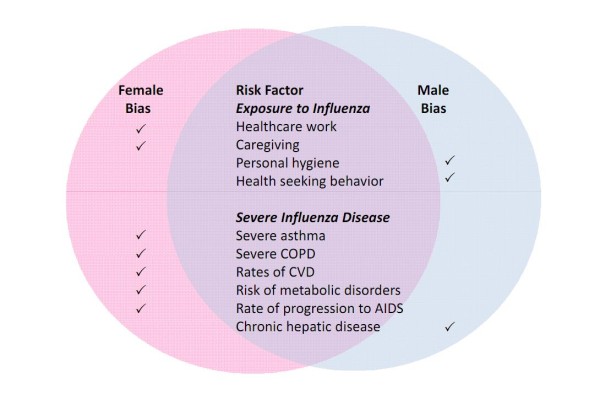
**Sex and gender biases in risk factors and co-morbidities for severe 2009 H1N1 disease**. Several risk factors predispose patients to increased morbidity and mortality from 2009 H1N1. The likelihood of engaging in behaviors associated with increased exposure as well as the severity and prevalence of co-morbidities associated with severe 2009 H1N1 disease differ between males and females.

Certain risk factors predispose patients to increased morbidity and mortality following exposure to influenza viruses [54] and the severity and prevalence of these underlying conditions often differ between males and females (Figure 4). The 2009 H1N1 virus causes disproportionate disease among young adults, a population that has a distinct repertoire of risk factors associated with exposure and worse outcome following infection compared with very young or old.

#### Occupational risk

Healthcare workers, as well as those in frequent contact with young children, are at a higher risk of exposure to influenza viruses than the general public [55]. Women represent over 50% of the healthcare workforce in many countries and nurses, teachers of young children and day-care workers are predominantly female [10] which potentially leads to a gender-specific occupational risk for influenza acquisition.

#### Personal hygiene

Hand hygiene compliance, one of the most effective ways to prevent transmission of influenza, is significantly better among female than male (male OR: 0.6, 95% CI: 0.4-0.98) healthcare workers [56]. Among healthcare workers in the USA, self-reported rates of use and knowledge about appropriate personal protective equipment in response to influenza are similar between the sexes [57].

#### Health seeking behaviour

Differences in health seeking behaviour or healthcare access may impact both the acquisition and manifestation of influenza. A WHO survey in 59 countries from 2002-2004 revealed that adult women are more likely to seek healthcare in both higher and lower income countries [10]. The quality of care for women in some parts of the developing world is not equal to that received by men [58]. In some developing countries, knowledge of the pandemic was higher among men than women, which might reflect the fact that there are greater educational opportunities and greater chances of socialization for men [59].

#### Chronic diseases

Chronic medical conditions predispose patients to increased influenza-related morbidity [54,60] and male-female, as well as pregnancy-associated, differences in disease prevalence have been reported.

##### (1) Respiratory disease

Asthma has been a significant underlying condition in children and adults hospitalized with critical illness [27,61]. Data from the USA and Canada illustrate that, prior to puberty, boys have more asthma exacerbations than girls. However, this trend is reversed in adulthood [39]. Rates of asthma attacks, numbers of asthma-related emergency room visits, numbers of asthma-related hospitalizations and duration of hospitalization are higher in women than men in the USA [39,62]. Rates of asthma, as well as incidence of asthma attacks, appear to be the same in pregnant and age-matched non-pregnant women [63].

Cystic fibrosis and COPD have been identified as risk factors for severe illness with 2009 H1N1 [3] and the progression of cystic fibrosis and long-term survival is significantly worse for females than males, especially among individuals diagnosed in childhood [64]. Females with COPD report worse symptoms, lower exercise capacity, more airway hyper-responsiveness and worse health-related quality of life than males [65,66]. Although morbidity from these conditions may be worse in females, mortality - both from all causes and from respiratory-related disease alone - is still higher in males with COPD [65], illustrating the complexities involved in assessing the significance of sex and gender for a particular co-morbidity.

##### (2) Hepatic disease

Chronic hepatic disease is a risk factor for severe 2009 H1N1 disease [3]. The development of hepatocellular carcinoma occurs at a 2:1 to 4:1 ratio for males to females [67]. The prevalence of serum hepatitis B virus (HBV) is consistently higher in men than women [68]. Males are more than twice as likely to die from liver cancer, which suggests that men may be more sensitive to the effect of HBV infection on the development of liver cancer [69]. Men also are twice as likely to develop cirrhosis [70].

##### (3) Cardiovascular disease

The rates and severity of cardiovascular disease differ between the sexes and these differences have been evaluated in the elderly [71]. As they have not been identified as an at-risk population for severe disease from 2009 H1N1 influenza, sex differences in cardiovascular disease may not be a critical factor.

##### (4) Metabolic disorders

Diabetes and morbid obesity have emerged as novel risk factors for severe 2009 H1N1 disease [3]. The lifetime risk of diabetes is higher in women than men, at least in the USA where approximately 55% of all diabetic-related deaths are women which may be a reflection of the fact that women tend to live longer than men [72]. In the USA, gestational diabetes and rates of diabetes in obese adolescent girls have been increasing [73,74]. Gestational diabetes occurs in up to 14% of all pregnancies [75]. Women, particularly those of lower socioeconomic status, also receive less adequate diabetes care than men of the same socioeconomic status [76].

Females, particularly in developing countries, tend to have higher rates of obesity [77]. According to the WHO, in 138 of 195 countries, females are over 50% more likely to be obese than males [78]. In some countries, the body mass index for women is 5-8 points higher than for men [78]. The higher rates of obesity and diabetes in females may be significant factors contributing to higher 2009 H1N1-related morbidity in women. It has not yet been determined whether pre-pregnancy obesity or excess weight gain during pregnancy represent equivalent risks. Precise parameters for documenting obesity in pregnant women have not been established [79].

#### *Immunocompromised individuals*

Influenza in immunocompromised individuals is associated with an increased severity of disease [54] and HIV is recognized as a co-morbidity for 2009 H1N1 influenza [54,80]. The rate of HIV in females are approaching that of males worldwide [81]. HIV RNA levels are consistently lower in women than men [82]. However, women have a 1.6-fold higher risk of progression to AIDS than men with equal viral loads [83,84]. There also are gender disparities in access to care for women with HIV, with women traditionally having greater difficulty accessing treatment [85,86]. Whether infection with HIV and progression to AIDS differentially affects the outcome of influenza virus infections in males and females has not been evaluated.

### Sex, gender and pregnancy effects on responses to influenza vaccines and antiviral therapies

The precise impact of sex, gender and pregnancy on responses to the 2009 H1N1 vaccines is not known [87-90]. Data from clinical trials of seasonal influenza vaccines reveal pronounced sex differences in the rates of vaccination, antibody responses to the vaccines and adverse reactions to the vaccines and illustrate that these differences must be considered in response to the 2009 H1N1 vaccine. Seasonal influenza vaccination data further reveal that pregnant and non-pregnant women generate comparable immune responses and experience similar adverse side effects [54].

#### Rates of vaccination

Available data on rates of 2009 H1N1 vaccination have not been analysed by sex [91] but rates of seasonal influenza vaccination vary significantly with respect to sex and age [92-94]. Rates of vaccination among women are lower than men in some European countries [92] and may reflect greater negative beliefs about the risks associated with vaccination [95], differences in physician recommendations regarding vaccination or occupational differences. Among healthcare workers in China, 73% of women reported intentions to decline both the H5N1 and 2009 H1N1 vaccines compared to 64% of men [96]. In France, acceptance (either receipt or intention to receive) of the 2009 H1N1 vaccine was higher among men and was higher among pregnant women and other groups with co-morbid conditions [97]. In the USA and Canada, vaccination against seasonal and 2009 H1N1 influenza during pregnancy is recommended irrespective of trimester [54,98,99]. The vaccination rate of pregnant women against 2009 H1N1 virus has been estimated to be only 38%, which is still higher than that normally seen with seasonal influenza vaccine [91].

#### Antibody responses to vaccines

Numerous studies reveal that haemagglutination inhibition (HAI) titres following seasonal influenza vaccination are consistently higher in women than men of comparable ages [100-104], which suggests that women may be better protected against influenza disease following vaccination than are men. Women aged 18-64 years generate a more robust neutralizing antibody response following vaccination than men [102]. Pregnant women appear to have similar responses to seasonal influenza vaccines compared to non-pregnant women. The National Institutes of Health reports that 47 out of 50 (94%) pregnant women immunized with 2009 H1N1 vaccine achieved antibodies levels considered to be protective within 21 days of inoculation [105].

#### Adverse reactions to vaccines

Women report more severe local and systemic reactions to influenza virus vaccines [100,102,104,106-108]. Women also experience worse reactions to vaccine adjuvants [109], which should be considered for 2009 H1N1 vaccines that are administered with adjuvant [88,89]. The extent to which adverse reactions to the 2009 H1N1 vaccine differ in either frequency or severity between males and females has not been reported [60].

Seasonal, H5N1 and MF-59-adjuvanted influenza vaccines are reported to be safe for pregnant women [110-112]. A study of 50 pregnant women who received the 2009 H1N1 vaccine reported it was well-tolerated with no significant adverse side effects documented [105].

#### Antiviral therapy

Antivirals are an effective treatment following infection with influenza viruses when administered early during the course of disease. The 2009 H1N1 viruses analysed, to date, are all resistant to the adamantadine class of antivirals but remain sensitive to neuraminidase inhibitors [3]. Available data indicate that the rate of prescribing antivirals to seasonal influenza virus-infected individuals, ranging in age from infants to adults, is similar between males and females in the USA [113-115]. In contrast, inappropriate prescription of antibiotics for seasonal influenza is greater for women [114]. A meta-analysis of data from randomized, double-blind clinical trials illustrates that, following treatment with oseltamivir, men return to their baseline wellness faster than women, suggesting that antiviral treatment for seasonal influenza may be more effective in men [116]. Whether this observation reflects patient reporting biases, need for differential drug doses or other confounding factors is not clear. These data do, however, indicate that sex and gender should be considered when evaluating the efficacy of antiviral treatment for 2009 H1N1.

Prompt administration of neuraminidase inhibitors is recommended for any pregnant woman with influenza-like symptoms [3]. Administration of antivirals within 48 h of symptom onset correlates with a mild or uneventful disease course in pregnant women [47,48,51]. Pregnant women who do not take antivirals, or begin treatment >72 h after symptom onset, have significantly higher morbidity and mortality rates compared to those who have early antiviral treatment [48,49].

### Sex differences in immune responses to viruses

Sex differences in the immune responses to influenza viruses have not been systematically examined [117]. Using data from other virus-host systems, several immunological, hormonal and genetic mechanisms have been identified as being differentially expressed between the sexes and altered during the course of pregnancy, which may account for male-female differences and pregnancy-associated increases in the severity of 2009 H1N1. Generally, women mount higher immune responses to viral infections [118]. Heightened antiviral immunity in women is beneficial for virus clearance, but may be detrimental if it becomes excessively high or prolonged, leading to pathology and even death. Over the course of pregnancy, inflammatory and antiviral immune responses are suppressed which can alter responses to viruses, such as influenza.

#### HIV

Women are at a greater risk of progressing to AIDS than men, despite having significantly less HIV RNA in circulation and host-mediated pathology is hypothesized to contribute to this sex bias [82]. Plasmacytoid dentritic cells (pDCs) are significant producers of type I interferons (IFN-α), which signal the activation of cytotoxic T cells for the elimination of virally infected cells. pDCs from women react more strongly to HIV-1 encoded toll-like receptor 7 (TLR7) ligands than pDCs derived from men, resulting in higher levels of immune cell activation [83]. Women with higher progesterone (P4) concentrations have greater numbers of activated pDCs in response to the HIV TLR7 ligand than women with lower P4 concentrations [83]. Several genes (for example, the *Tlr7 *gene that encodes a receptor that recognizes RNA viruses, including influenza viruses) that encode for immunological proteins are on the X chromosome and may escape X inactivation, resulting in higher amounts of expression in women [117]. X chromosomal variation also alters the course of progression of AIDS differently in women than men [84]. Whether female-biased immunopathology contributes to the severity of 2009 H1N1 disease in women requires consideration.

#### Hepatitis B virus

The prevalence of HBV, titres of HBV DNA and development of hepatocellular carcinoma are higher in males than females and involve the effects of hormones on viral and host gene expression [67,68,119]. Among HBV positive males, elevated concentrations of testosterone and expression of certain androgen receptor gene alleles correlate with an increased risk of hepatocellular carcinoma [120,121]. In HBV transgenic mice, castration of males reduces, whereas replacement of testosterone in castrated males increases, serum HBsAg concentrations [122]. Chemically-induced hepatocellular carcinoma is more severe in male than female mice, which is mediated by increased inflammatory cytokine production by liver cells in males and can be reversed with oestradiol (E2) treatment [123]. Sex steroids modulate sex differences in the prevalence of HBV and development of liver cancer through effects on immune responses to HBV. Whether sex steroids affect the pathogenesis of influenza virus infection should be examined.

#### Sex steroids and immunity

The impact of sex steroids, including androgens, oestrogens and progesterone (P4), on the activity of immune cells may contribute to sex differences and the effects of pregnancy on responses to 2009 H1N1. Generally, androgens, including dihydrotestoesterone and testosterone, suppress the activity of immune cells [124]. The immunosuppressive effects of androgens may reflect the inhibitory effects of androgen receptor signalling mechanisms on transcriptional factors that mediate the production of pro-inflammatory and antiviral cytokines [125].

Oestrogens affect both innate and adaptive immune function. Oestradiol can have bipotential effects with low doses enhancing and high doses reducing proinflammatory cytokine production [126]. Low E2 concentrations promote helper T cell type 1 (Th1) responses and cell-mediated immunity and high concentrations of E2 augment helper T cell type 2 (Th2) responses and humoral immunity which may be responsible for some female as well as pregnancy-associated changes in immune responses [126].

Another oestrogen that affects the functioning of the immune system is oestriol (E3), which is produced during pregnancy by the placenta. When E3 levels are high, inflammatory responses and the symptoms of Th1-mediated autoimmune diseases - including multiple sclerosis - are reduced [127,128]. Whether the effects of pregnancy on responses to 2009 H1N1 reflect the effects of E3 on immune responses requires investigation.

Progesterone suppresses innate immune responses [125,129]. Elevated concentrations of P4 during pregnancy inhibit the development of Th1 immune responses that can lead to fetal rejection and promote production of Th2 immune responses [130,131]. Progesterone also suppresses antibody production [132]. Recent data illustrate that pregnant women with severe 2009 H1N1 have lower levels of total IgG2 than healthy pregnant women or women with only moderate H1N1 disease [133]. As IgG2 levels are enhanced in a Th1-dependent manner, this reduction in total IgG2 may be related to pregnancy-associated modulation of the immune response.

## Conclusions

As data from the pandemic continue to be analysed, a number of factors should be considered by clinicians, epidemiologists and scientists in order to better understand the role of sex, gender and pregnancy on 2009 H1N1 disease.

• Age- and sex-associated differences in exposure and severity of infection must be documented, as many biological and behavioural differences occur over the course of the lifespan.

• The outcome of infection is worse for females, but the magnitude of this difference varies across countries and the differential contribution of gender and sex in different regions of the world must be considered.

• Excessively high innate and cell-mediated immune responses, including the production of cytokines and chemokines, may contribute to increased severity of influenza in females.

• Higher antibody responses to influenza vaccines in females may lead to an increased protection from disease.

• Sex should be considered when effective vaccine and antiviral dosages are determined in order to maximize efficacy while limiting adverse side effects.

• As the outcome of influenza infection can be worse for females, efforts should be made to increase acceptance of vaccines in both pregnant and non-pregnant females.

• The 2009 H1N1 infection of pregnant women needs to be studied carefully in order to determine the factors that are driving the increased morbidity and mortality rates.

• Sex hormones have profound effects on the immune responses to vaccines and infection and should be examined in clinical samples and animal models.

• Animal models of infection can provide important insights into the role of sex, pregnancy, and hormones on the immune response to vaccination, infection, and antiviral treatment.

## Abbreviations

CI: confidence interval; COPD: chronic obstructive pulmonary disease; DC: dendritic cell; E2: 17β-oestradiol; E3: oestriol; HBV: hepatitis B virus; OR: odds ratio; P4: progesterone; pDC: plasmacytoid DC; RR: relative risk; Th1: helper T cell type 1; Th2: helper T cell type 2.

## Competing interests

The authors declare that they have no competing interests.

## Authors' contributions

SLK, CP, MA, PO and AP extensively discussed, researched and outlined the paper. SLK and AP drafted the manuscript and figures and edited the manuscript. CP, MA and PO edited the manuscript. All authors read and approve the final manuscript.
